# MGMT assessment in pituitary adenomas: comparison of different immunohistochemistry fixation chemicals

**DOI:** 10.1007/s11102-018-0862-x

**Published:** 2018-01-17

**Authors:** Alexander S. G. Micko, Romana Höftberger, Adelheid Wöhrer, Matthias Millesi, Engelbert Knosp, Stefan Wolfsberger

**Affiliations:** 1 Department of Neurosurgery, Medical University Vienna, Waehringer Guertel 18-20, 1097 Vienna, Austria; 2 Institute of Neurology, Medical University of Vienna, Vienna, Austria

**Keywords:** Pituitary adenoma, MGMT, Time dependent, Promoter methylation

## Abstract

**Purpose:**

Despite the established role of O6-methyl-guanine-DNA methyltransferase (MGMT) as a marker for temozolomide response, consensus of the most reliable method to assess MGMT expression in pituitary adenomas is still missing. Currently, immunohistochemistry (IHC) assessment of formaldehyde fixed tissue samples is most widely used in a semiquantitative description. As formaldehyde fails to completely preserve nucleic acids, RCL2, an alcohol-based formaldehyde-free fixative, has been proposed as a more reliable alternative in terms of cell stability. Furthermore, as the current method of IHC is semiquantitative and observer-dependent, pyrosequencing, an objective tool to evaluate the methylation status of the MGMT promoter, has emerged as a reliable and accurate alternative. The aim of this study was to validate the current IHC method for assessment of MGMT protein expression in pituitary adenomas.

**Methods:**

The tissue samples of 8 macroadenomas with positive IHC MGMT expression (> 50%) were investigated: first, we compared the time dependent stability of MGMT protein expression after pituitary adenoma removal between formaldehyde vs. RCL2. Then, we compared positive IHC MGMT expression with methylated promoter status using pyrosequencing.

**Results:**

In the first 12 h after adenoma removal, tissue samples remained MGMT positive in significantly more samples when fixated with formaldehyde than with RCL2, respectively (96 vs. 81%, p = 0.025).

**Conclusion:**

Our data confirm that the current method using formaldehyde tissue fixation and IHC reveals stable and reliable results of MGMT assessment in pituitary adenomas.

## Introduction

Aggressive pituitary adenomas and carcinomas pose a treatment challenge because they often remain incurable despite multiple surgeries, endocrine therapy and radiation treatment.

Temozolomide (TMZ), an alkylating agent, which is the mainstay of treatment for high-grade gliomas and advanced melanoma [1–4], has also shown effectiveness against aggressive pituitary adenomas and carcinomas [5–12].

A positive response to TMZ has been found in association with downregulation of the DNA repair protein O6-methyl-guanine-DNA methyltransferase (MGMT) which removes alkylating adducts induced by TMZ and counteracts its antineoplastic action [6, 9, 11–22]. MGMT is a ubiquitously present protein in human cells but the amount of expression varies greatly within normal tissues. It is located on chromosome 10q26 and consists of 207 amino acids with a molecular mass of 21,645 Da [23–26] in human cells. The half-life of the mRNA as estimated in different cell lines is approximately 10 h [27, 28].

Despite the established role of MGMT as a possible marker for TMZ response, consensus of the most reliable method to assess MGMT expression in pituitary adenomas is still missing. Currently, immunohistochemistry (IHC) assessment of formaldehyde fixed tissue samples is most widely used in a semiquantiative description [6, 9, 11, 12, 15, 21]. Differences in methodology of MGMT immunostaining and assessment however, complicate the comparison of existing studies thus the clinical value of MGMT expression for pituitary tumours [12, 29, 30].

However, formaldehyde fails to completely preserve nucleic acids, and RCL2, an alcohol-based formaldehyde-free fixative, has been proposed as a more reliable alternative in terms of cell stability [31–33]. Furthermore, the current method is semiquantitative and observer-dependent. Pyrosequencing, an objective tool to evaluate the methylation status of the MGMT promoter which detects pyrophosphate release on nucleotide of the next complementary nucleotide, has emerged as a reliable and accurate alternative in other tumor types [34].

The aim of this study was to validate the current IHC method for assessment of MGMT protein expression in pituitary adenomas.

## Materials and methods

### Patient series

We evaluated 8 MGMT positive pituitary adenomas of a consecutive series of 16 patients with macroadenomas (diameter > 1 cm) at the immediate time point of tumor tissue removal. The tissue of these operations was divided into two equal parts, one for neuropathological examination the other for research purposes. The tumor samples were evaluated by the consent of the patients for further histopathological examination (EC Nr:1008/2014).

### Histopathologic examinations

Immediately after surgical removal, each tumor sample was divided into 18 pieces of at least 2 mm^3^ size. In a time dependent mode (at 0 min, 30 min, 1 h, 2 h, 6 h, 12 h after operation) one piece was fixed in 4.5% neutral buffered formaldehyde and one in RCL2, respectively at each time. Furthermore, one piece was frozen in liquid nitrogen in case of possible unclear results or necessary evaluation at a later date.

All samples were embedded in paraffin, cut at 5 μm and stained with both hematoxylin and eosin and the periodic acid-Schiff method. Paraffin-embedded tissue sections were examined immunohistochemically using the mouse monoclonal antibodies, MGMT (Ab-1, Clone MT 3.1; Waltham, Massachusetts, USA; 1:50 dilution). Staining was performed with a Ventana BenchMark ULTRA (Ventana Medical Systems Inc., Tucson, Arizona, USA) automated immunostainer.

After deparaffinization, 5 μm thick sections have undergone heat-induced epitope retrieval in citrate buffer, pH 6.0. Tissue sections were incubated overnight at 4 °C with the primary antibody. The next day, sections were labelled with the appropriate secondary antibody, incubated with avidin-biotin-peroxidase, and visualized with a standard diaminobenzidine (DAB) detection kit (Ventana iVIEW DAB Universal Kit). Sections were then counterstained with Mayer’s hematoxylin.

Positive control tissues for IHC consisted of paraffin-embedded sections of colon cancer for immunostaining of MGMT. A positive internal control was done by staining of vascular endothelial cells. We used a non-relevant antibody of the same species (mouse) and of the same immunoglobulin isotype (IgG1) as negative control.

### Assessment

For MGMT protein expression evaluation, each tumor sample was classified as: < 10, 10–25, 25–50, 50–75 and > 75% immunopositive cells [9, 35]. According to their clinical relevance these groups were further stratified to the two distinguished groups, < 50% (*negative* and *intermediate* immunoexpression), > 50% (*positive* immunoexpression) as proposed [35, 36].

The immunoreactivity of MGMT was evaluated under light-microscopy at ×20–40 magnification by three observers (A.M., A.W. and R.H) in a randomized and blinded mode. Only areas with highest immunoreactivity and minimal necrosis, fibrosis or other artifacts were selected for evaluation.

### Pyrosequencing

For pyrosequencing analysis, 5 μm slices were cut from the formaldehyde stained and paraffin embedded blocks and were investigated at the Institute of Cancer Research, Medical University of Vienna. DNA isolation was performed using the EpiTect FFPE Lysis Kit (Qiagen, Hilden, Germany) according to manufacturer’s recommendations.

For definition of methylated/unmethylated MGMT promoter, the percentage mean value of the four investigated CpG dinucleotides (genomic sequence on chromosome 10 from 131,265,519 to 131,265,537:CGACGCCCGCAGGTCCTCG) was calculated.

A cut-off percentage of mean methylation, due to clinical relevance, was determined at 8% (< 8% MGMT promoter unmethylated; ≥ 8% MGMT promoter methylated) as previous publications described for glioblastomas [34, 37, 38].

### Statistical analysis

To assess differences within formaldehyde and RCL2 samples χ^2^ test was used. The same method was chosen to evaluate differences between IHC and pyrosequencing formaldehyde fixated samples.

A p-value < 0.05 was considered significant. For statistical analyses SPSS® version 23.0 software (SPSS Inc., Chicago, IL, USA) has been used.

## Results

The tissue samples of 8 macroadenomas with positive IHC MGMT expression (> 50%) were investigated. The histological examination showed that there were 4/8 null-cell adenomas and 4/8 gonadotropinomas.

First, we compared the stability of MGMT protein expression after pituitary adenoma removal in formaldehyde vs. RCL2 in a time dependent mode. Then, we compared positive IHC MGMT expression with methylated promoter status using pyrosequencing.

### Formaldehyde vs. RCL2

Overall eight pituitary adenoma samples fixated at six different time points, 46/48 (96%) formaldehyde cases remained positive (MGMT expression > 50%), while 39/48 (81%) cases showed stable results in the RCL2 group (p = 0.025).

**Fig. 1:**
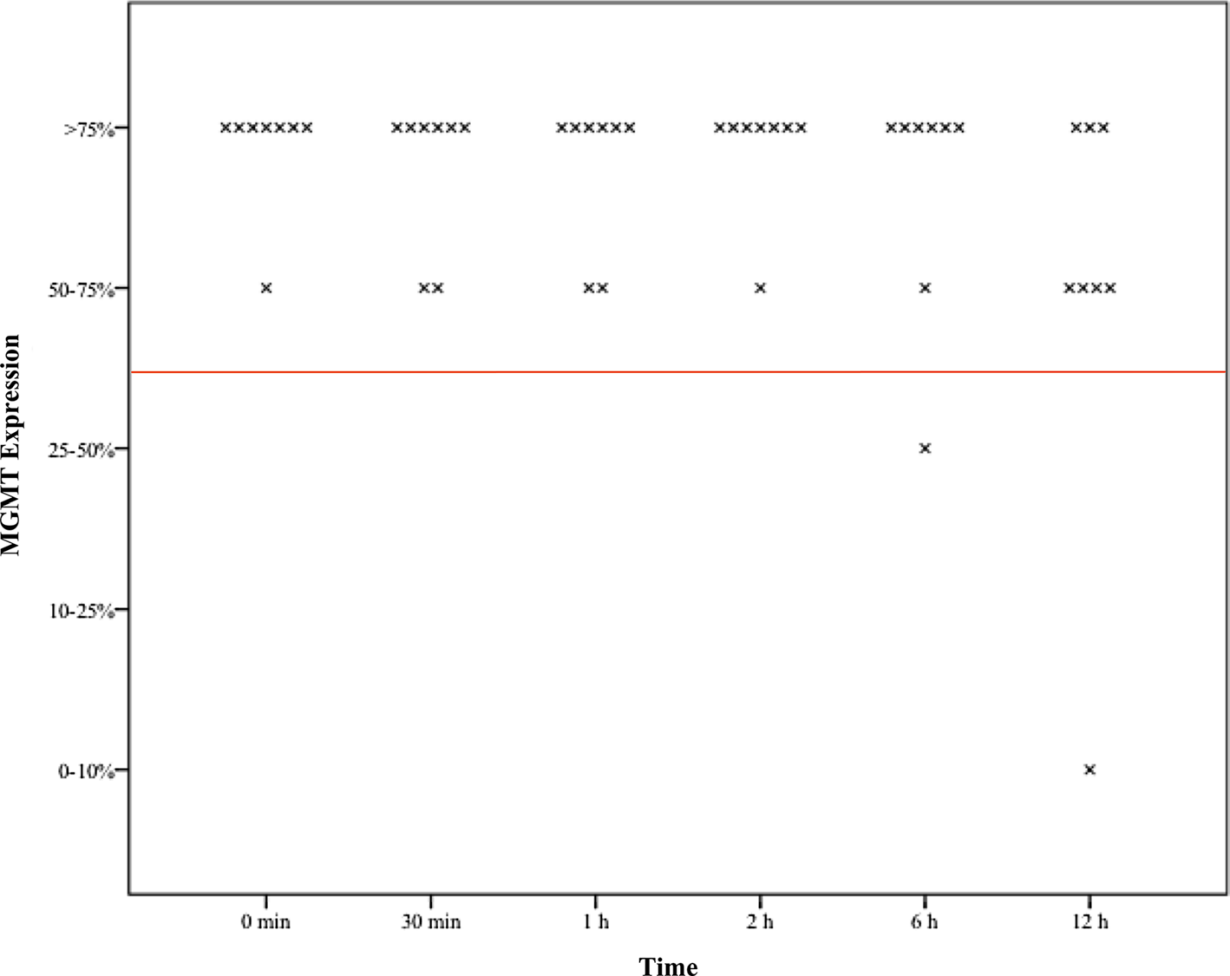
Boxplots of assessed time dependent MGMT expression in formaldehyde fixed tissue samples x—demonstrating the respective MGMT expression to the defined time point red line—demonstrating values above and below 50% MGMT Expression

**Fig. 2:**
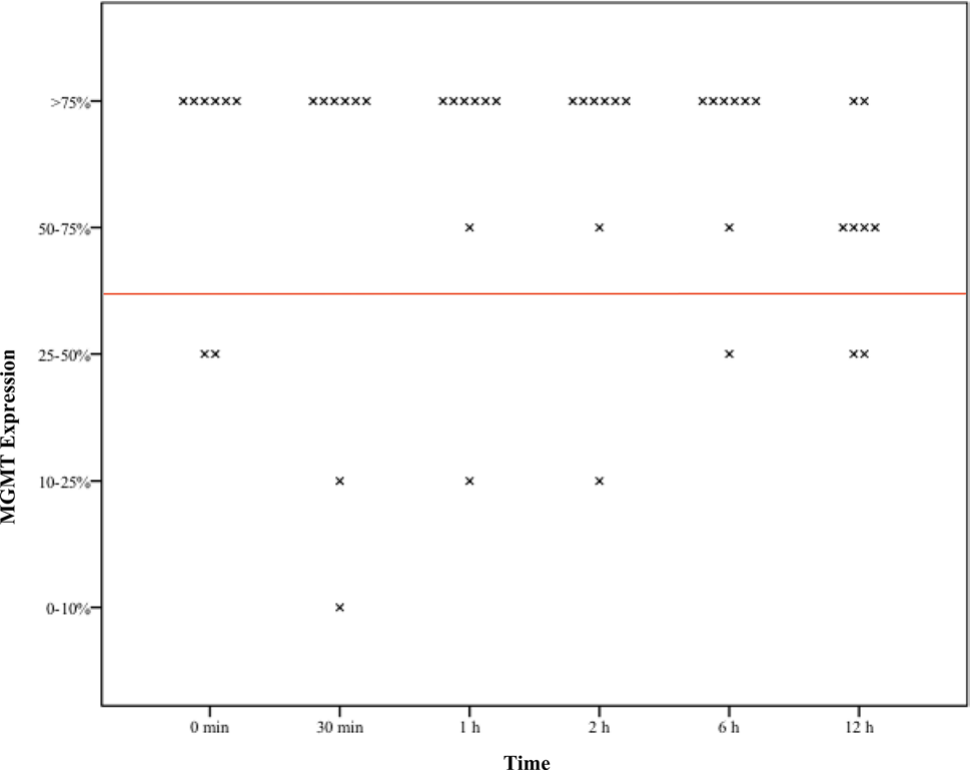
Boxplots of assessed time dependent MGMT expression in RCL2 fixed tissue sample x—demonstrating the respective MGMT expression to the defined time point red line—demonstrating values above and below 50% MGMT expression

In one patient MGMT continued to be positive during the whole investigation period whereas in RCL2 samples every sample showed negative (< 50%) results (p = 0.001). In one patient MGMT remained positive in 5/6 cases whereas all samples of RCL2 showed positive results (p = 0.296). In another patient, 5/6 formaldehyde samples were positive, however 2/5 RCL2 samples were positive (p = 0.376) (Table 1) (Figs. 1, 2).

**Table 1:** Comparison of IHC MGMT expression > 50%, formaldehyde vs. RCL2

Patient	Formaldehyde > 50%	RCL2 > 50%	p
Patient 1	6/6	6/6	NS
Patient 2	6/6	0/6	0.001
Patient 3	6/6	6/6	NS
Patient 4	5/6	4/6	NS
Patient 5	6/6	6/6	NS
Patient 6	5/6	5/6	NS
Patient 7	6/6	6/6	NS
Patient 8	6/6	6/6	NS
Total	46/48	39/48	0.025

*NS* non significant (p > 0.05)

### Pyrosequencing

**Fig. 3:**
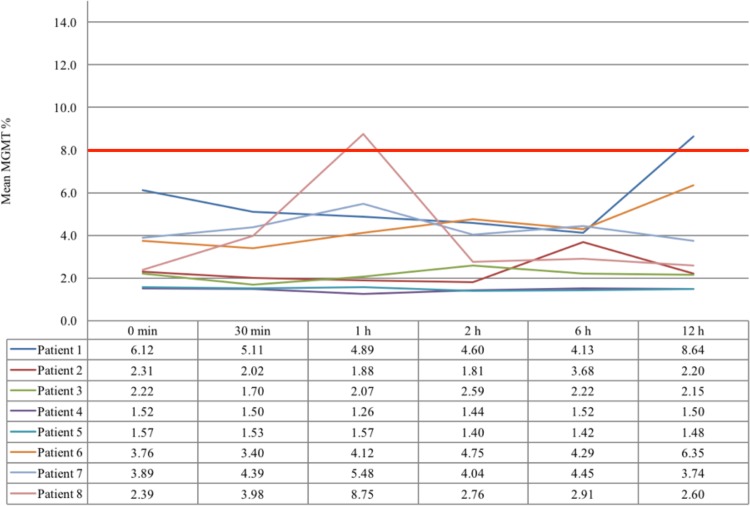
Time dependent MGMT pyrosequencing results bold red line—demonstrating values above and below 8% MGMT promotor methylation

We observed an unmethylated MGMT promoter in all adenoma tissue samples immediately after the operation (1.52–6.12%). In 2/48 (4%) cases the MGMT promoter changed to methylated (defining a cut-off for methylated MGMT promoter ≥ 8%) in one patient after 1 h and in another patient after 12 h after tumor removal. MGMT pyrosequencing results are shown in detail in Fig. 3.

## Discussion

Aggressive pituitary adenomas and pituitary carcinomas unresponsive to multiple surgeries, drug treatment and radiotherapy pose a considerable therapeutic challenge. To date, chemotherapy with the alkylating drug TMZ has been the most effective treatment alternative for approximately half of these patients [5–13, 30, 36, 39–46].

In clinical routine, negative MGMT expression was found the most reliable predictive marker for tumor response to TMZ. However, MGMT expression was found to not correlate with biological tumor behavior and TMZ treatment success in every case (positive response to TMZ in only 73% of MGMT negative cases) [12]. In this respect, we validated the time-dependent stability of MGMT expression with the currently performed tissue fixation and assessment against alternative methods.

### Pyrosequencing

Pyrosequencing, a relatively new technique compared to IHC, has proven stable interobserver results in promoter methylation analysis of glioblastoma samples and thus has found its way into clinical routine setting [34]. Furthermore, methylation of the CpG islands of the MGMT promoter has shown to correlate with loss of MGMT protein expression in tumor tissue [47]. This finding was also shown in pituitary adenomas using methylation analysis of the MGMT promoter. However, the frequency of methylation is considerably lower in pituitary tumors than in glioma cell lines [11]. Furthermore, pyrosequencing of promoter methylation in tumor samples can be contaminated with MGMT positive normal cells like leukocytes and endothelial cells and therefore maybe show false positive results [48–51].

A definitive cut-off for MGMT promotor methylation at the CpG position of 8% in pituitary adenomas, like in glioma cell lines, has not been defined to date. We applied the same criteria to the adenoma samples in our present series and revealed no statistical significant difference compared to MGMT positive IHC tissue samples.

However, comparing the costs between IHC and pyrosequencing at our institute, examinations revealed that taken into account only the pure costs for material (without acquisition or staff costs), the charges for one sample pyrosequencing were 63.5€ vs. 2.3€ for one IHC sample.

### Immunostaining of MGMT

Differences in the technique of fixation, preservation and duration of paraffin-embedding have been reported to alter results in immunostaining of MGMT [11]. IHC has the advantage that technical expertise and equipment is widely available, in contrast to pyrosequencing. Furthermore, IHC allows differentiating between adenoma cells and non-neoplastic cells as well as identification of heterogeneity within tumor samples.

A relatively small variability of MGMT expression within a given pituitary adenoma has been attributed to the homogeneous population of adenoma cells in contrast to more heterogeneous glioma cells [52].

### Time of adenoma tissue fixation

**Fig. 4:**
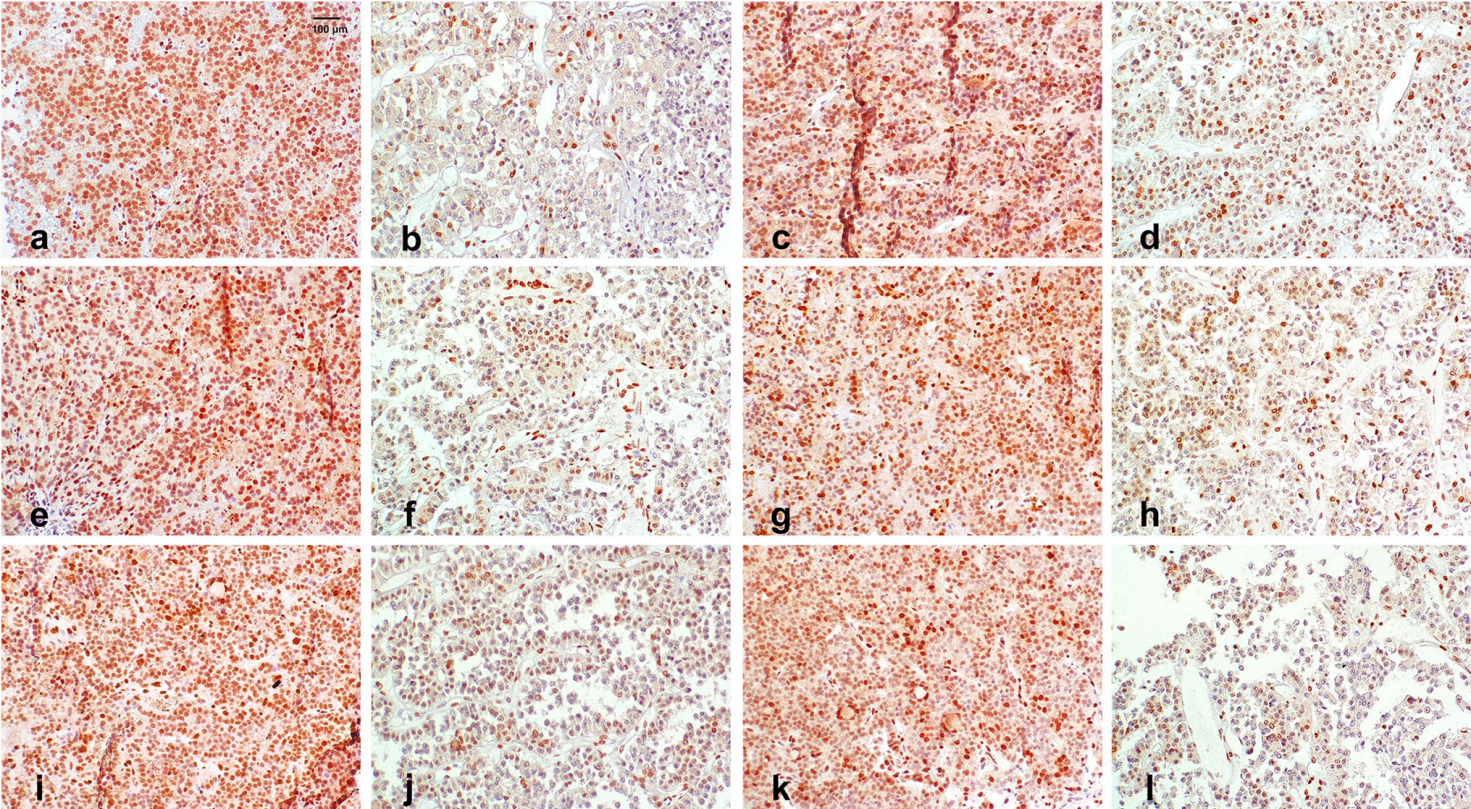
Case 2: time dependent MGMT expression, ×40 magnification (**a, b**) time point = 0 min after tumour removal (**a** formalin > 75%; **b** RCL2 0–10%), **c, d** time point = 30 min after tumour removal (**c** formalin 50–75%; **d** RCL2 10–25%), **e, f** time point = 1 h after tumour removal (**e** formalin 50–75%; **f** RCL2 10–25%), **g, h** time point = 2 h after tumour removal (**g** formalin > 75%; **h** RCL2 10–25%), **i, j** time point = 6 h after tumour removal (**i** formalin > 75%; **j** RCL2 25–50%), **k, l** time point = 12 h after tumour removal (**k** formalin 50–75%; **l** RCL2 25–50%)

Furthermore, false negative results of MGMT expression may be due to different time points of fixation after tissue removal. The positive MGMT expression of neuron and glia cells has been found to decrease and vanish after exitus [53, 54]. We therefore investigated tissue samples over a time period of 12 h of 8 patients in detail to investigate a possible decrease of MGMT expression. We found that in formaldehyde fixation samples the results remained positive (IHC MGMT expression > 50%) in 96% in formaldehyde fixed samples (Fig. 4).

## Conclusion

In conclusion, our data confirm that the current method using formaldehyde tissue fixation and IHC reveals stable and reliable results of MGMT assessment in pituitary adenomas.
